# Influence of support surfaces on the distribution of body interface pressure in surgical positioning*

**DOI:** 10.1590/1518-8345.2692.3083

**Published:** 2018-11-29

**Authors:** Karoline Faria de Oliveira, Patrícia da Silva Pires, Ana Lúcia De-Mattia, Elizabeth Barichello, Cristina Maria Galvão, Cleudmar Amaral de Araújo, Maria Helena Barbosa

**Affiliations:** 1 Universidade Federal do Triângulo Mineiro, Departamento de Enfermagem na Assistência Hospitalar, Uberaba, Minas Gerais, Brazil.; 2 Universidade Federal da Bahia, Escola de Enfermagem, Salvador, Bahia, Brazil; 3 Universidade Federal de Minas Gerais, Escola de Enfermagem, Belo Horizonte, Minas Gerais, Brazil; 4 Universidade de São Paulo, Escola de Enfermagem de Ribeirão Preto, PAHO/WHO Collaborating Centre for Nursing Research Development, Ribeirão Preto, SP, Brazil.; 5 Universidade Federal de Uberlândia, Faculdade de Engenharia Mecânica, Uberlândia, Minas Gerais, Brazil.

**Keywords:** Patient Positioning, Patient Safety, Perioperative Care, Perioperative Nursing, Perioperative Period, Pressure Ulcer

## Abstract

**Objective:**

to evaluate the interface pressure (IP) of support surfaces (SSs) on bony prominences.

**Method:**

a quasi-experimental study with repeated measures on each SS. Twenty healthy adult volunteers participated in the study. The participants were placed in the supine position on a standard operating table for evaluation of IP on the bony prominences of the occipital, subscapular, sacral, and calcaneal regions using sensors. Seven evaluations were performed for each bony prominence: one on a standard operating table, and the others on tables containing SSs made of viscoelastic polymer, soft foam, or sealed foam. Descriptive statistics and analysis of variance were used to analyze the data.

**Results:**

the mean IP was higher on the viscoelastic polymer-based SS compared to the other SSs (p<0.001). The mean IP was relatively lower on the density-33 sealed foam and density-18 soft foam. In addition, this variable was comparatively higher in the sacral region (42.90 mmHg) and the calcaneal region (15.35 mmHg).

**Conclusion:**

IP was relatively lower on foam-based SSs, especially on density-18 soft foam and density-33 sealed foam. Nonetheless, IP was not reduced on the viscoelastic polymer SS compared to the control SS.

## Introduction

Support surfaces (SSs) are specialized devices, overlays, pads, and integrated systems that redistribute body pressure. These devices are designed to control pressure, shearing, and fabric friction while maintaining the microclimate or other therapeutic functions^1^.

The redistribution of body pressure, especially on bony prominences, is the primary safety characteristic of positioning materials^2^, which aim to prevent complications such as pressure ulcers (PU)^3^ and compartment syndrome^4^.

The etiology of PU involves, among other factors, interface pressure (IP), characterized by compression of soft tissues between the bony prominences and the surfaces on which patients lie. Exposure to IP over prolonged periods decreases tissue perfusion and oxygenation of the skin and deeper layers. In view of this causal relationship, the present study used IP as a criterion for assessing PU risk^5^ ^-^ ^8^.

The literature does not indicate an acceptable threshold for IP. However, there is evidence that the mean capillary refill pressure is 32 mmHg, and this criterion was adopted for evaluating IP^5^ ^-^ ^8^ because the external pressure that exceeds this level may obstruct blood flow. IP was evaluated on various bony prominences using SSs made of foams, gels, polyurethane, and polyethylene ^5^ ^-^ ^8^.

There are gaps in knowledge on the behavior of SSs in the redistribution of IP because of delays in technological advancements in health^7^, methodological limitations, and lack of standardization in classifying SSs^1^. Few studies to date determined the IP redistribution of these materials in the surgical setting.

The objective of this study is to evaluate the IP of SS [viscoelastic polymer, sealed foams (28, 33, and 45 kg m^3^), and soft foams (18 and 28 kg m^3^)] on the bony prominences of the occipital, subscapular, sacral, and calcaneal regions.

The viscoelastic polymer was selected because it is a static SS highly recommended for clinical surgical practice^8^ and is frequently used as a test surface in laboratory studies^5^. Sealed and soft foams of different densities were selected because of their potential as raw materials for producing lower-cost SSs; therefore, they may be a more cost-effective alternative for redistributing pressure on bony prominences. The density that best distributes IP should be evaluated to provide evidence that support decision-making for purchasing SSs.

## Methods

This preliminary and interdisciplinary quasi-experimental study was conducted in two partner research centers located in two public universities in the Triângulo Mineiro region, state of Minas Gerais, Brazil, and specialized in two distinct areas of research: nursing and mechanical engineering. Measurements were performed in the research center specialized in mechanical engineering using high-precision equipment and software, and clinical evaluation was performed by the core nursing research team.

The study protocols complied with the guidelines established by the Revised Standards for QUality Improvement Reporting Excellence (SQUIRE 2.0)^9^.

The participants were non-randomly selected from the academic community of the university in which data were collected to field this study by invitation to volunteer. The initial invitation was made by e-mail sent to potential participants. The message contained information about the study objectives, the importance of participation, and the risks and benefits of participation.

The inclusion criteria were being older than 18 years and the presence of chronic comorbidities as long as these were controlled. The exclusion criteria were the presentation of skin lesions, impairment of bony prominences, absence of limbs, or presence of folds in the limbs.

The literature does not present the parameters for calculating the sample size for assessing IP. Therefore, an initial sample of 20 participants was selected, and statistical power was analyzed later. A significance level of 0.05 was adopted for estimating statistical power.

Statistical power was estimated for differences in mean IP using different SSs. A power of 99% was reached within the limits of the statistical program’s precision. In clinical and practical terms, there was a difference in maximum IP between the SSs, which justified not including more participants in the study.

The research was conducted in a large public teaching hospital in the state of Uberaba, Minas Gerais, Brazil. Data were collected in April 2017 on the weekends (Saturday and Sunday) in the morning, afternoon, and night, and during workdays at night because none of the scheduled surgeries were performed in these periods. The data were collected by a Ph.D. student after receiving training in anthropometric measurement and IP evaluation.

The study participants were sent to the hospital’s anthropometry room to be evaluated according to the inclusion and exclusion criteria. The objective of the study was clarified, and each participant signed an informed consent form.

The participants were asked to undress and put on a hospital gown open on the back and specifically designed for the study. The weight and height of the participants were measured, and body mass index (BMI) was calculated by dividing the weight in kilograms by the square of the height^10^.

Weight was measured using a Filizola analog scale with a precision of 0.1 kg. The participants were weighed barefoot, standing, with their arms hanging alongside the body.

Height was measured using a vertical stadiometer scaled in centimeters and millimeters. The participants were positioned on the scale barefoot, heels together, and feet forming a 45° angle, in an upright position, with eyes fixed on the horizon. Readings were made to the nearest centimeter when the horizontal rod of the vertical bar on the scale touched the participant’s head^10^.

The nutritional status was determined according to guidelines of the World Health Organization (WHO)^10^ as follows: underweight, BMI < 18.5 Kg/m^2^; normal weight, BMI of 18.5-24.9 Kg/m^2^; overweight, BMI of 24.9-29.9 Kg/m^2^; and obese, BMI > 29.9 Kg/m^2^. Five participants from each nutritional status were selected.

The participants were assessed for standard procedures adopted in the hospital. In typical situations, this involves positioning the patient on a standard operating table (SOT). The patient was placed on the SOT in the supine position, covered with a cotton sheet, with the upper limbs supported by supine clamps. No SS was added between the SOT and the patient. The SOT was a Barrfab surgical table (212 cm × 59 cm) containing a foam mattress covered with a waterproof lining. IP on the SOT is considered the control measurement.

It should be pointed out that all IP evaluations were performed in a sterile surgical suite of the hospital’s surgical center. The surgical suite had a Barrfab SOT, and air conditioning to control room temperature and relative humidity to ensure that the conditions for our patients were the same as those for patients subjected to anesthetic-surgical procedures.

The participants were placed on the SOT and measurements were made on each SS, totaling 20 evaluations for each group. The following SSs were evaluated: viscoelastic polymer (Akton), sealed foam density 28 kg/m^3^(D28) (Luckspuma), density 33 kg/m^3^(D33) sealed foam (Luckspuma), sealed foam density 45 kg/m^3^(D45) (Luckspuma), soft foam density 18 kg/m^3^(D18) (Luckspuma), and soft foam density 28 kg/m^3^(D28) (Luckspuma).

The dimensions of the viscoelastic polymer were 183.0 cm x 50.0 cm x 1.3 cm, and the manufacturer reported that this product did not require a cover made of other materials. The dimensions of the sealed (D28, D33, D45) and soft (D18, D28) foams were 212 cm x 59 cm, with a thickness of 5 cm. These SSs were protected with a cotton cloth (surgical table sheet), which was exchanged after evaluating each participant.

IP was measured using a mesh of sensors, the CONFORMat system (Tekscan^®^). This system uses a Windows-based software and includes a thin and flexible sensor consisting of 1,024 sensing elements to measure IP in a tissue area of 530 mm x 617 mm.

The sensing elements are arranged in rows and columns in the sensor mesh. The software uses a map to convert the pressure detected by the hardware into pressure data and correctly display the sensor output in the window in real time. The sensor had been previously calibrated for use with each SS. At the time of assessment, the calibrations were changed for each SS.

IP was evaluated in each bony prominence region (occipital, subscapular, sacral, and calcaneal). It should be pointed out that the experiment involved evaluations of all the SSs in this study. These regions were selected because of their higher rate of PU in the supine position^11^.

**Figure 1:**
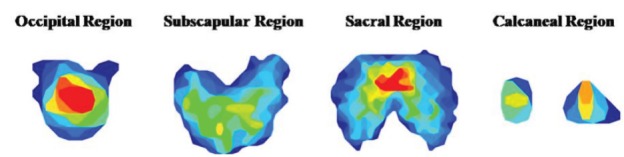
Detection frames of the occipital region, subscapular region, sacral region, and calcaneal region. Uberaba, Minas Gerais, Brazil, 2017

For measuring each body prominence, the volunteers remained in the supine position for one minute, which was the time required to complete the film of the image’s detection frames (Figure 1).

The participants were asked to indicate when they were relaxed before starting film recording and not to move or speak during measurements. The mean peak pressure values were determined in millimeters of mercury (mmHg).

Before placing the participant on the CONFORMat sensor, the adequacy of the positioning and distribution of the sensors was checked to ensure they were under the regions to be evaluated. Measurements were made along the caudal-cephalic axis because of the size of the sensor and were initiated in the occipital and subscapular regions. The participant was repositioned when necessary, and the sensor was placed in the sacral region and then in the calcaneal region. Therefore, the images were acquired in three steps for each SS.

An instrument created by the researchers was used to collect sociodemographic, anthropometric, and IP data. This instrument was subjected to validation of appearance and content by five evaluators with experience in this field of study. These data were entered into Excel spreadsheets and, after double data entry and validation, were analyzed using the Statistical Package for Social Sciences software version 20.0 for Windows.

The qualitative variables (types of SS and nutritional status) were analyzed by descriptive statistics using absolute frequencies, percentage distributions, and contingency tables. For the quantitative variables (age, BMI, and mean peak pressure), descriptive measures of centrality (mean) and dispersion [standard deviation (SD)] and minimum and maximum values were used.

Analysis of variance (ANOVA) of repeated measures for a single factor was used to assess statistically significant differences between the SSs for the pressure exerted on the occipital, subscapular, sacral, and calcaneal bony prominences. For numerical variables, repeated-measures ANOVA for multiple factors was used to verify statistically significant differences according to nutritional status (underweight, normal weight, overweight, and obese). The level of significance was 5%.

This study was approved by the Research Ethics Committee of the Federal University of Triângulo Mineiro (Protocol No. 48855615.6.0000.5154) in accordance with the precepts of National Health Council Resolution 466/2012 of the Ministry of Health of Brazil.

## Results

The mean age of the study participants was 28.2 years, ranging from 19.0 to 59.0 years. Most of the study sample were women (90%). The minimum BMI was 16.73 Kg/m^2^, with a maximum of 44.96 Kg/m^2^ and a mean of 25.85 Kg/m^2^.

**Figure 2:**
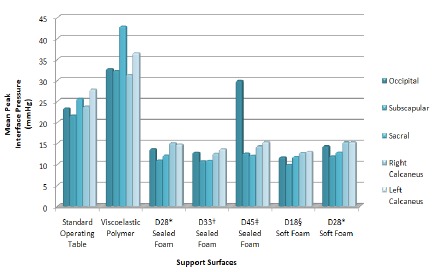
Distribution of the mean peak interface pressure in the occipital, subscapular, sacral, right calcaneal, and left calcaneal regions on different support surfaces. Uberaba, Minas Gerais, Brazil, 2017

The mean peak IP was relatively higher on all bony prominences on the viscoelastic polymer SS compared to the other materials and the SOT (Tables 1 and 2 and Figure 2).

**Table 1:** Distribution of the means, standard deviations, and minimum and maximum peak interface pressure in the occipital, subscapular, sacral, right calcaneal, and left calcaneal regions on different support surfaces. Uberaba, Minas Gerais, Brazil, 2017

Region	Mean peak interface pressure (mmHg)		Support surfaces						
			SOT*	Viscoelastic polymer	Density 28 sealed	Density 33 sealed	Density 45 sealed	Density 18 soft	Density 28 soft
Occipital	F^†^= 31.76 p^§^ = 0.001	Mean	23.40	32.80	13.65	12.80	29.94	11.70	14.35
		SD^‡^	5.43	7.80	3.39	3.91	15.29	3.26	4.42
		Minimum	15.00	22.00	10.00	9.00	9.00	9.00	7.00
		Maximum	33.00	48.00	25.00	26.00	23.00	21.00	24.00
Subscapular	F^†^= 34.83 p^§^ = 0.001	Mean	21.65	32.30	11.00	10.80	12.60	9.95	11.95
		SD^‡^	12.14	12.82	3.58	5.36	3.10	4.06	4.85
		Minimum	12.00	12.00	7.00	7.00	9.00	5.00	7.00
		Maximum	68.00	63.00	22.00	31.00	20.00	21.00	22.00
Sacral	F^†^= 53.87 p^§^ = 0.001	Mean	25.65	42.90	12.15	10.90	12.10	11.80	12.85
		SD^‡^	9.83	17.45	1.66	2.71	2.59	2.39	3.18
		Minimum	14.00	24.00	9.00	6.00	10.00	7.00	9.00
		Maximum	48.00	94.00	16.00	18.00	20.00	16.00	23.00
Right calcaneus	F^†^= 33.87 p^§^ = 0.001	Mean	23.80	31.35	15.10	12.55	14.35	12.75	15.30
		SD^‡^	8.63	12.77	4.35	3.46	3.83	3.75	4.59
		Minimum	7.00	16.00	6.00	7.00	8.00	7.00	8.00
		Maximum	45.00	60.00	24.00	21.00	24.00	21.00	27.00
Left calcaneus	F^†^= 41.37 p^§^ = 0.001	Mean	27.85	36.55	14.75	13.65	15.35	13.05	15.30
		SD^‡^	9.09	14.52	3.68	2.85	3.27	3.56	4.21
		Minimum	11.00	19.00	8.00	6.00	10.00	7.00	9.00
		Maximum	47.00	77.00	22.00	19.00	24.00	19.00	24.00

*SOT, standard operating table; ^†^F, analysis of variance of repeated measures for a single factor; ^‡^SD, standard deviation; ^§^p, p-value

**Table 2:** Interface pressure in the occipital, subscapular, sacral, right calcaneal, and left calcaneal regions using different support surfaces according to the analysis of variance. Uberaba, Minas Gerais, Brazil, 2017

Region	Support surfaces	SOT*	Viscoelastic polymer	D28^†^ sealed	D33^‡^ sealed	D45^§^ sealed	D18^ǁ^ Soft	D28^†^ soft
Occipital	SOT*	-	<0.001	<0.001	<0.001	1.00	<0,001	<0,001
	Viscoelastic polymer	<0.001	-	<0.001	<0.001	1.00	<0.001	<0.001
	Sealed D28^†^	<0.001	<0.001	-	1.00	0.03	0.12	1.00
	Sealed D33^‡^	<0.001	<0.001	1.00	-	0.02	1.00	0.66
	Sealed D45^§^	1.00	1.00	0.03	0.02	-	0.001	0.007
	Soft D18^ǁ^	<0.001	<0.001	0.12	1.00	0.001	-	0.13
	Soft D28^†^	<0.001	<0.001	1.00	0.66	0.007	0.13	-
Subscapular	SOT*	-	0.022	0.030	0.015	0.071	0.003	0.011
	Viscoelastic polymer	0.022	-	<0.001	<0.001	<0.001	<0.001	<0.001
	Sealed D28^†^	0.030	<0.001	-	1.00	1.00	1.00	1.00
	Sealed D33^‡^	0.015	<0.001	1.00	-	1.00	1.00	1.00
	Sealed D45^§^	0.071	<0.001	1.00	1.00	-	0.102	1.00
	Soft D18^ǁ^	0.003	<0.001	1.00	1.00	0.102	-	0.084
	Soft D28^†^	0.011	<0.001	1.00	1.00	1.00	0.084	-
Sacral	SOT*	-	<0.001	<0.001	<0.001	<0.001	<0.001	<0.001
	Viscoelastic polymer	<0.001	-	<0.001	<0.001	<0.001	<0.001	<0.001
	Sealed D28^†^	<0.001	<0.001	-	0.368	1.00	1.00	1.00
	Sealed D33^‡^	<0.001	<0.001	0.368	-	0.398	1.00	0.009
	Sealed D45^§^	<0.001	<0.001	1.00	0.398	-	1.00	1.00
	Soft D18^ǁ^	<0.001	<0.001	1.00	1.00	1.00	-	1.00
	Soft D28^†^	<0.001	<0.001	1.00	0.009	1.00	1.00	-
Right calcaneus	SOT*	-	0.057	0.001	<0.001	<0.001	<0.001	0.006
	Viscoelastic polymer	0.057	-	<0.001	<0.001	<0.001	<0.001	<0.001
	Sealed D28^†^	0.001	<0.001	-	0.425	1.00	0.363	1.00
	Sealed D33^‡^	<0.001	<0.001	0.425	-	1.00	1.00	0.027
	Sealed D45^§^	<0.001	<0.001	1.00	1.00	-	1.00	1.00
	Soft D18^ǁ^	<0.001	<0.001	0.363	1.00	1.00	-	0.492
	Soft D28^†^	0.006	<0.001	1.00	0.027	1.00	0.492	-
Left calcaneus	SOT*	-	0.041	<0.001	<0.001	<0.001	<0.001	<0.001
	Viscoelastic polymer	0.041	-	<0.001	<0.001	<0.001	<0.001	<0.001
	Sealed D28^†^	<0.001	<0.001	-	1.00	1.00	1.00	1.00
	Sealed D33^‡^	<0.001	<0.001	1.00	-	0.089	1.00	0.651
	Sealed D45^§^	<0.001	<0.001	1.00	0.089	-	0.293	1.00
	Soft D18^ǁ^	<0.001	<0.001	1.00	1.00	0.293	-	0.587
	Soft D28^†^	<0.001	<0.001	1.00	0.651	1.00	0.587	-

*SOT, standard operating table; ^†^D28, density 28; ^‡^D33, density 33; ^§^D45, density 45; ^|^D18, density 18

*D28, density 28; †D33, density 33; ‡D45, density 45; §D18, density 18

The mean peak IP was comparatively lower on the D33 sealed foam and D18 soft foam compared to the other materials (Table 1 and Figure 3) and the SOT (Table 2).

The mean IP was relatively higher in the sacral and left calcaneal regions using the viscoelastic polymer, corresponding to 42.90 and 36.55 mmHg, respectively.

The mean IP was higher in the calcaneal region on the D28 and D33 sealed foam, and D18 and D28 soft foams. Moreover, this variable was highest in the left calcaneal and sacral regions on the SOT.

There were no statistically significant differences in the mean peak IP using the D45 sealed foam compared to the SOT in the occipital and subscapular regions (Table 2).

A multivariate, multiple-factor analysis was performed to assess differences in the mean peak IP between the study groups according to nutritional status (underweight, normal weight, overweight, and obese). There were no significant differences between the groups (p=0.87) (Table 3).

**Table 3:** Means, standard deviations, and minimum and maximum peak interface pressure in the sacral region on different support surfaces according to nutritional status. Uberaba, Minas Gerais, Brazil, 2017.

Variables	Nutritional status		SOT*	Viscoelastic polymer	Density 28 sealed	Density 33 sealed	Density 45 sealed	Density 18 soft	Density 28 Soft
Mean peak interface pressure (mmHg) F^†^=0.29 p^‡^=0.87	Underweight	Mean	29.80	42.80	10.60	8.20	11.20	9.40	11.80
		Standard deviation	14.81	29.07	1.14	1.30	0.84	1.82	1.48
	Normal weight	Mean	25.40	41.60	12.20	12.40	13.20	11.40	12.80
		Standard deviation	11.63	13.05	1.30	3.36	3.96	1.52	3.77
	Overweight	Mean	24.00	45.00	12.40	10.00	11.20	12.20	12.00
		Standard deviation	7.65	18.71	1.14	0.71	1.30	0.84	1.22
	Obese	Mean	23.40	42.20	13.40	13.00	12.80	14.20	14.80
		Standard deviation	3.85	8.53	1.95	1.73	3.11	2.49	4.82

*SOT, standard operating table; ^†^F, analysis of variance of repeated measures for multiple factors; ^‡^p, p-value.

## Discussion

The precise measurement of IP depends on several factors, including equipment calibration and the proper use and number of sensing elements per tissue area. A higher number of sensing elements per tissue area may increase measurement sensitivity. The number of sensors per tissue area in the equipment used in the present study was higher than that in other studies that used pressure mapping technologies^5^ ^-^ ^6^ ^,^ ^12^ ^-^ ^13^.

An experimental study in Belgium mapped IP on different SSs using the ErgoCheck System detection technology, which is composed of 684 sensors^5^. A cross-sectional study performed in a university hospital in Sweden used the Mapping System, with four sensors in a mesh of 45 cm x 45 cm ^12^. A study conducted in the United States used the XSensor System, with a square resolution of 0.25 inches for an extension of 48 inches x 48 inches^6^. Therefore, the technologies used for areas of detection by sensors were inferior to that used in the present study.

An experimental study that evaluated the pressure distribution properties of an electrophysiology laboratory surface and an operating room table used the FSA Mapping System, which is a mesh of 1,024 sensors with a detection area of 1920 mm x 762 mm^13^. Although the number of sensors was the same as that used in the present study, the detection area of this system was 4.5 times larger, which might affect measurement sensitivity.

A study conducted in the United States evaluated mean IP in the supine position using an electro-pneumatic sensor^14^; nonetheless, this study provided no information about the dimensions of the sensor and other specifications, which limited comparisons between the technologies used.

With respect to the immobilization time of the participants to measure IP values, the methodology proposed in this study followed that of other studies, whereby immobilization time did not alter the pressure detected by the sensors^5^ ^,^ ^15^.

Mean IP was relatively higher on the viscoelastic polymer SS compared to other foams and the SOT. Studies with different research designs and outcomes did not recommend the use of viscoelastic polymers or indicated that evidence was not sufficient to make a recommendation^16^ ^-^ ^18^.

It should be pointed out that differences in nomenclature of some SSs may create confusion about the materials used across studies. For instance, in the experimental study conducted in Belgium^5^, the viscoelastic polymer was designated gel SS.

An integrative review carried out by the Wound, Ostomy, and Continence Nurses Society also observed inconsistencies in the terminology for SS^1^, indicating the need to standardize the nomenclature because differences in terminology hamper comparisons between studies.

IP was significantly lower for sealed and soft foams than the control group, and peak IP was lowest for D18 soft foam and D33 sealed foam. IP was lower for D28 sealed foam and D33 sealed foam relative to D28 soft foam. However, differences in IP between sealed and soft foams were not statistically significant.

The Belgian study found that foam mattresses had little or no effect on pressure reduction, and therefore these mattresses did not effectively prevent PU^5^, and this result differs from that of the present study.

The results of a study conducted in an integrated hospital in the southeast United States showed that 85% of patients with PU used devices in the form of foam pillows. The authors inferred that the high incidence of PU could be related to the use of obsolete SS^19^.

Another study conducted in the United States compared mean IP in the subscapular, sacral, and calcaneal regions on two SSs made of a three-layer common foam and high-density foam (3.5 inches). The results indicated that there were no significant differences between the tested SSs. Mean IP in the sacral region was higher than capillary refill pressure (37.51 mmHg and 38.18 mmHg, respectively)^14^. These results do not agree with our findings, in which mean IP on different types of foam was lower than capillary refill pressure.

In a cross-sectional study in the United States, the foams used were not fully characterized. Furthermore, the authors used SSs with overlapping layers, which compromised comparisons between studies^14^.

A study conducted in Belgium compared IP on four SSs relative to the SOT, including gel SS (Action^®^), a 3-cm foam SS, a viscoelastic polyether SS (SAF^®^), and a viscoelastic polyurethane SS (Tempur-Pedic^®^). IP was significantly lower on the gel SS relative to the SOT (43.6 mmHg and 49.2 mmHg, respectively)^5^. These results do not agree with ours, in which IP was higher on the viscoelastic polymer SS compared to the SOT.

A cross-sectional study conducted in Sweden evaluated peak IP on four SSs: an SOT made of high-strength polyurethane (50 kg/m^3^), a high-resilience foam mattress with pressure redistribution (50-52 kg/m^3^), an air-filled mattress (not supplied air) with an outer viscoelastic foam layer, and a 188-mm thick alternating pressure mattress. Peak IP on the SOT was 64.1 mmHg^12^. These results differ from ours, in which peak IP was relatively lower.

An experimental study evaluated the pressure distribution between a 2.5-inch surface (Tempur-Pedic^®^ EP) made of viscoelastic material (Tempur-Pedic North America, Inc, Lexington, KY) and a 4-inch viscoelastic surface (Medline Industries, Inc, Mundelein, IL). The highest IP recorded by the sensors on the 4-inch viscoelastic surface was 90 mmHg^13^. In the present study, the highest IP in the sacral region on the viscoelastic polymer SS was 94 mmHg.

The results of the present study indicated that IP was comparatively higher in the sacral and calcaneal regions on the viscoelastic polymer SS and the SOT, which corroborates the conclusions of a retrospective chart review that evaluated the factors contributing to the development of PU in patients who underwent surgical procedures^19^.

An experimental study found that mean peak IP was higher in the sacral region on the Eggcrate^®^ SS compared to the SOT (59 ± 17 mmHg, p=0.01) and a gel mattress (61 ± 27 mmHg, p=0.02). On the heels, mean peak IP was lower on Eggcrate (70 ± 24 mmHg) compared to the SOT (122 ± 58 mmHg, p=0.02) and the gel mattress (134 ± 59 mmHg, p=0.005)^6^. IP on the SOT was higher than the value found in the present study.

In the calcaneal region, the results of a study conducted in the United States indicated that pressure on the heel was high on most SSs^6^, which agrees with our findings and indicate the need to implement actions to relieve this pressure when this body region is elevated.

There were no statistically significant differences in IP between the groups according to nutritional status. It is important to consider that nutritional status is a useful evaluation criterion adopted by many researchers but expresses only a relationship between two variables (body weight and height). In this respect, individuals with the same nutritional status may have different body compositions (relationship between lean body mass, fat mass, and body water volume), which may explain the absence of correlation between BMI and IP.

A previous study found a positive relationship between body composition and IP and proposed a virtual reference model for the action of tension on the analyzed tissue. In this study, the stress caused by IP was more evident in the muscle layer. Furthermore, there was no relationship between the fat layer and a higher level of muscle shearing^20^.

In view of differences in research findings, it is necessary not only to evaluate IP but also to consider that ulcer etiology has multiple causes, including tissue tolerance to pressure and shearing, and this tolerance may be affected by microclimates (heat and humidity), nutrition, perfusion, associated diseases, and tissue condition^3^. Body composition is also relevant because different types of tissue have distinct reactions to pressure.

One of the limitations of the present study is the participation of healthy volunteers. Although data were collected in environmental conditions similar to those to which surgical patients are exposed, some factors related to the procedure should be considered. Anesthesia and patient clinical status affect the body’s hemodynamics and are risk factors for PU. Furthermore, surgical procedures involve adding operative fields and surgical manipulation, which may increase pressure in certain areas. Another study limitation was that most participants were women because IP distribution can be influenced by the deposition of adipose tissue in different regions. However, it should be noted that, although these issues were not considered, the purpose of the study was achieved.

The results of this study provide evidence that may help the clinical and managerial practice of nurses in choosing SSs that best redistribute IP on surgical tables during perioperative positioning. These findings demonstrate the importance of developing new products in this area of research because most of the products currently available are imported and expensive, which often makes their acquisition unviable considering the economic and social diversity of Brazil.

Further research is needed to evaluate the effect of microclimates on the etiology of PU using larger samples and individuals with different body compositions.

## Conclusion

Foam-based materials, specifically D33 sealed foam, redistributed body interface pressure more effectively on operating tables, and these promising results may stimulate the development of improved and cheaper support surfaces. Further clinical studies are necessary to evaluate the performance of these materials.
